# Supplementary figures and images for: Overexpression of miR-125b in Osteoblasts Improves Age-Related Changes in Bone Mass and Quality through Suppression of Osteoclast Formation
Source: Int J Mol Sci. 2021 Jun 23;22(13):6745. doi: 10.3390/ijms22136745 (PMC8267655; doi:10.3390/ijms22136745)

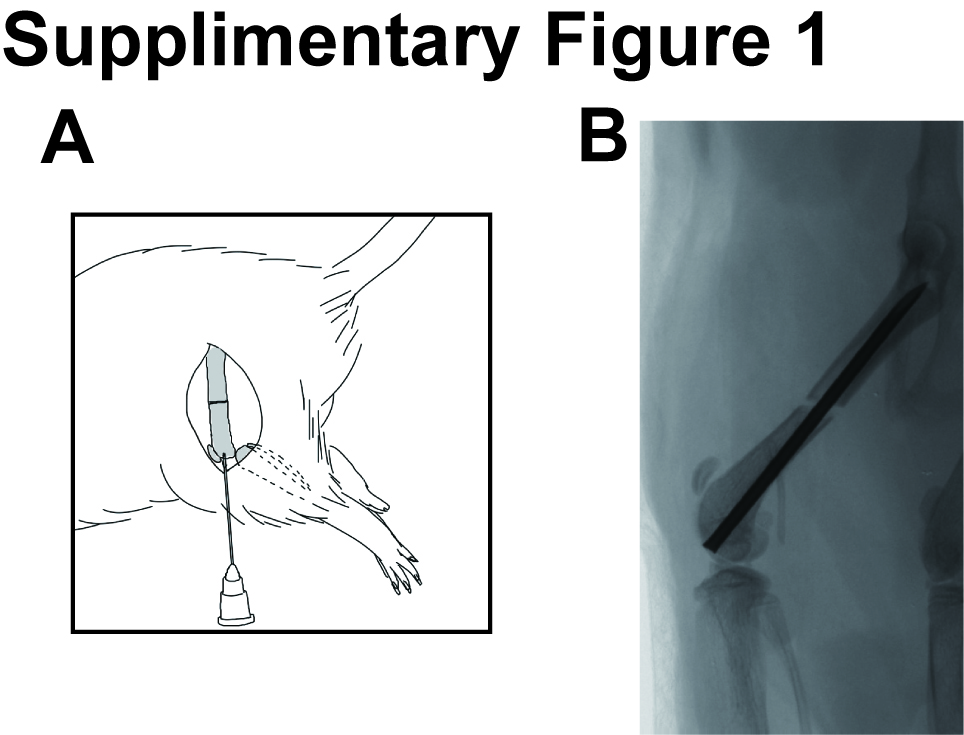

Supplement: Supplementary file 1 [file ijms-22-06745-s001.zip › Suppl Fig. 1.jpg]

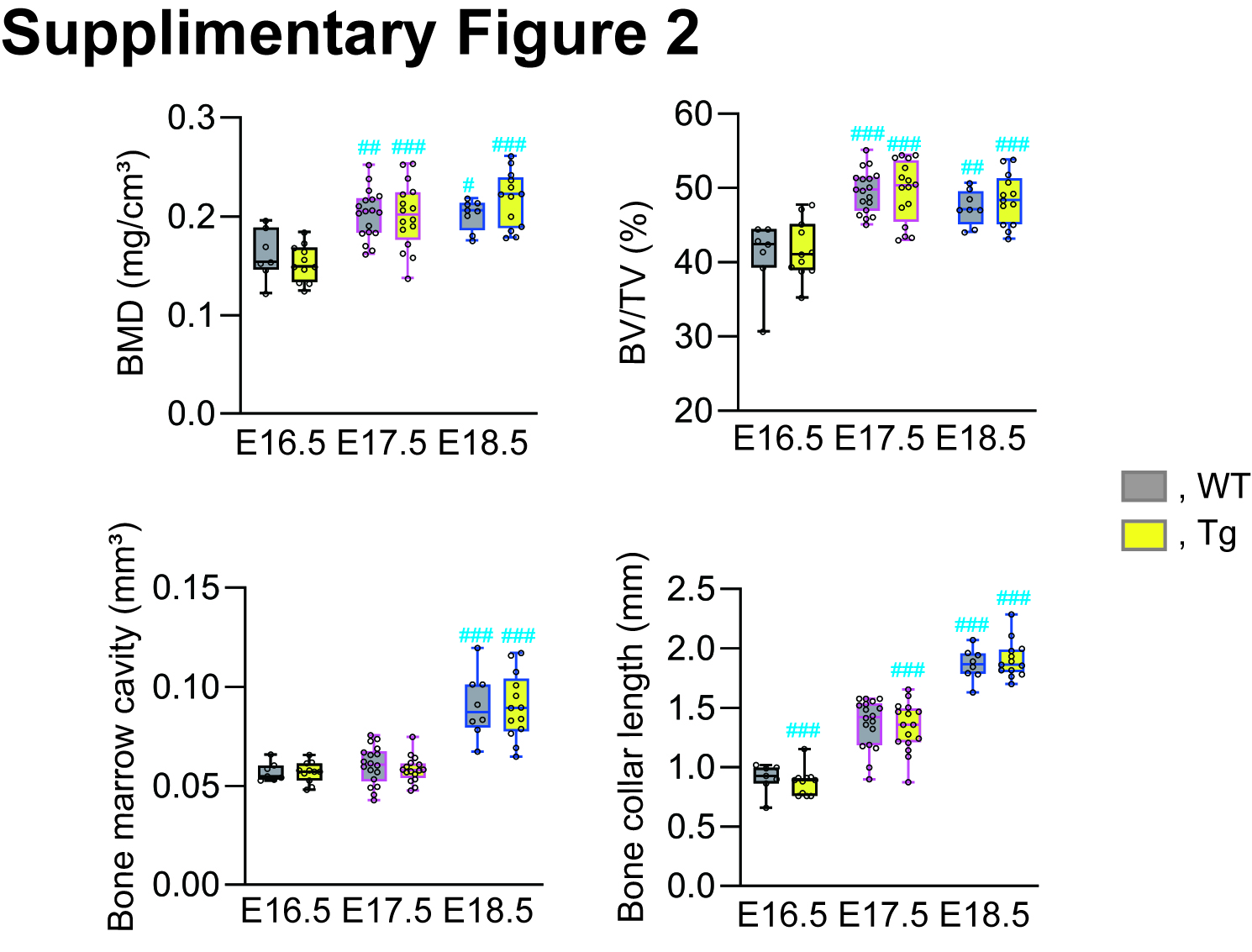

Supplement: Supplementary file 1 [file ijms-22-06745-s001.zip › suppl fig. 2.jpg]
